# Upregulation of iNOS/NO in Cancer Cells That Survive a Photodynamic Challenge: Role of No in Accelerated Cell Migration and Invasion

**DOI:** 10.3390/ijms25115697

**Published:** 2024-05-23

**Authors:** Albert W. Girotti, Witold Korytowski

**Affiliations:** 1 Department of Biochemistry, Medical College of Wisconsin, Milwaukee, WI 53226, USA; 2 Department of Biophysics, Jagiellonian University, 31-007 Krakow, Poland; witold.korytowski@uj.edu.pl

**Keywords:** malignant tumors, photodynamic therapy, nitric oxide, nitric oxide synthase, cancer cell proliferation, migration, invasion, bystander effects, PDT adjuvants

## Abstract

Anti-tumor photodynamic therapy (PDT) is a unique modality that employs a photosensitizer (PS), PS-exciting light, and O_2_ to generate cytotoxic oxidants. For various reasons, not all malignant cells in any given tumor will succumb to a PDT challenge. Previous studies by the authors revealed that nitric oxide (NO) from inducible NO synthase (iNOS/NOS2) plays a key role in tumor cell resistance and also stimulation of migratory/invasive aggressiveness of surviving cells. iNOS was the only NOS isoform implicated in these effects. Significantly, NO from stress-upregulated iNOS was much more important in this regard than NO from preexisting enzymes. Greater NO-dependent resistance, migration, and invasion was observed with at least three different cancer cell lines, and this was attenuated by iNOS activity inhibitors, NO scavengers, or an iNOS transcriptional inhibitor. NO diffusing from PDT-targeted cells also stimulated migration/invasion potency of non-targeted bystander cells. Unless counteracted by appropriate measures, all these effects could seriously compromise clinical PDT efficacy. Here, we will review specific examples of these negative side effects of PDT and how they might be suppressed by adjuvants such as NO scavengers or inhibitors of iNOS activity or expression.

## 1. Introduction: Pro-Tumor Effects of Nitric Oxide

Many malignant tumors exploit nitric oxide (NO), a free radical molecule, to promote tumor cell survival, proliferation, and overall motility [1,2,3,4]. These effects are typically observed at relatively low steady state levels of enzymatically generated NO, e.g., 10–100 nM. Three possible nitric oxide synthase (NOS) isoforms could give rise to this NO: neuronal (nNOS/NOS1), inducible (iNOS/NOS2), and endothelial (eNOS/NOS3). The iNOS isoform is most often associated with cancer initiation and progression, and its level in resected tumors is now considered a reliable prognostic indicator for patients with the highest levels given the lowest survival chances [5,6]. The most prominent cellular sources of the NO might be proximal macrophages or fibroblasts, or even tumor cells themselves, which we will focus on in this review. This is now clear from abundant experimental evidence, e.g., from (i) genetic ablation or knockdown of NOS enzyme(s), (ii) suppression of NOS activity with chemical inhibitors, (iii) inhibition of NOS transcription, or (iv) use of chemical agents that scavenge NO [1,2,3,4,5,6]. Among the several known effects of endogenous NO on tumor cells compared with normal counterparts are (i) elevated resistance to chemotherapy or radiotherapy, (ii) greater resistance to apoptotic cell death, (iii) increased angiogenesis, and (iv) greater migratory/invasive aggressiveness [1,2,3,4,5,6]. As emphasized in this review, many of these negative effects are exacerbated in tumor cells that can withstand an oxidative challenge, viz. one induced by anti-tumor photodynamic action.

## 2. Anti-Tumor Photodynamic Therapy

**Figure 1:**
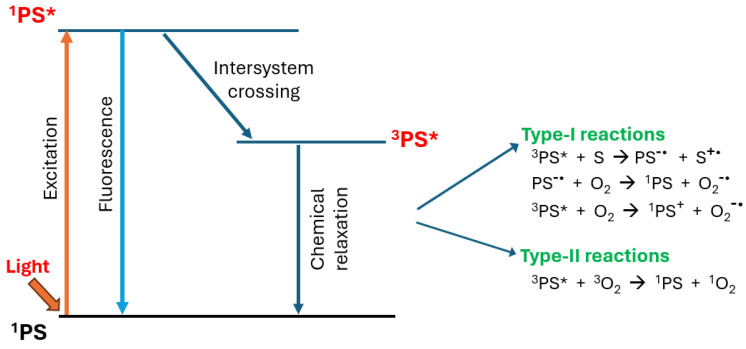
Type-1 and Type-2 mechanisms of photodynamic action in PDT.

Anti-tumor photodynamic therapy (PDT) was introduced about 50 years ago as a novel clinical approach for eradicating a variety of solid malignancies (e.g., bladder, breast, and head-neck) via cytotoxic photochemistry [7]. To generate cytotoxic reactive oxygen species (ROS), PDT requires (i) a photosensitizing agent (PS), (ii) PS photoexcitation by light in the far visible-to-near infrared wavelength range, and (iii) molecular oxygen [8,9,10]. All three components must be engaged concurrently to activate the photodynamic process, and light delivery via fiber optic systems makes PDT highly selective for an intended tumor target. Thus, PDT is essentially non-invasive, and compared with conventional chemotherapy or radiotherapy, exhibits little, if any, off-target cytotoxicity. A Type-2 PDT mechanism involves energy transfer from the photoexcited triplet state PS to ground state O_2,_ converting the latter to highly reactive singlet molecular oxygen (^1^O_2_), a non-radical ROS [11,12] (Figure 1). In contrast, a Type-1 mechanism involves electron transfer from triplet state PS to O_2_, resulting in the formation of oxyradical ROS such as superoxide (O_2_^−●^), and hydroxyl (HO^●^) [11,12] (Figure 1).

^1^O_2_ and oxyradicals can kill tumor cells by oxidizing vital proteins, nucleic acids, or lipids if they lie close to generation sites of these short-lived ROS. Solid tumors such as head-and-neck, bladder, pancreatic, and prostate have been treated by PDT using PSs such as Photofrin^®^ (the first to obtain FDA approval), benzoporphyrin derivative (BPD), and silicon phthalocyanine (Pc4) [8,9]. In addition to these active PSs, which are administered as such, pro-PSs have been developed which are converted to their active forms in recipient tumors. The most prominent example is 5-aminolevulinic acid (ALA), which is metabolized to protoporphyrin IX (PpIX) via the heme biosynthetic pathway [13,14]. This pathway tends to be more active in transformed cells than in normal counterparts, which at least partially explains why PpIX accumulates to higher levels in the former [14]. Mitochondria are the initial sites of this PpIX build-up, and its photoactivation can result in cell death via intrinsic apoptosis, which is initiated by mitochondrial damage, as discussed below [15]. Before proceeding, it is important to point out that the abbreviation PDT for “photodynamic therapy” usually represents action at the in vivo clinical level. However, PDT can also stand for “photodynamic treatment” when applied to action on isolated tissues or cells in vitro. A proper distinction should be made; thus, referring to in vitro treatment as “photodynamic therapy”, which occurs quite often, is incorrect.

## 3. Peroxidizable Membrane Lipids as PDT Targets: Antioxidant Effects of NO

Like other amphiphilic PSs of PDT relevance, ALA-induced PpIX typically localizes at membrane–aqueous interfaces, with the pyrrole ring methyl and vinyl groups projecting into the membrane, and propionic acid groups projecting into the aqueous phase [16]. Membrane interaction promotes PpIX’s ability to photosensitize peroxidation of unsaturated membrane lipids such as phospholipids, glycolipids, and cholesterol [12,17]. Although photodamage to membrane proteins can also occur, unsaturated lipids are more prominent targets due to their greater overall abundance. Lipid peroxidation (LPO) can be set off by ROS such as HO^●^ from Type-1 photochemistry or ^1^O_2_ from Type-2 photochemistry, although the latter is more prevalent with ALA-induced PpIX as PS [14]. In contrast to an oxyradical like HO^●^, which abstracts a lipid hydrogen to initiate free radical-mediated (chain) LPO, ^1^O_2_ adds directly to a nearby unsaturated lipid to give a lipid hydroperoxide (LOOH) with hydrogen retention and an allylic shift of the double bond [18]. Such LOOHs have been detected and quantified at ultra-high sensitivity/specificity using high-performance liquid chromatography with mercury cathode electrochemical detection [HPLC-EC(Hg) [19]. In the presence of suitably chelated Fe(III) and a reductant like NADH or ascorbate, membrane LOOHs can undergo 1-electron reduction to oxyl radical (LO^●^) or epoxyallylic peroxyl radical (OLOO^●^) intermediates which abstract hydrogen from other lipids, thereby triggering chain LPO [18,20]. Although this process may commence during irradiation, it is not light-dependent, and the resulting membrane damage may far exceed that due to prior photodynamic action alone. Chain LPO from 1-electron turnover of primary photogenerated LOOHs can persist long after irradiation is terminated, thus indicating a certain “momentum” in this post-irradiation process. Initial evidence for this after-light LPO was provided by observing the inhibitory effects of chain-breaking antioxidants like butylated hydroxytoluene, α-tocopherol, and chemical donor-derived NO at low levels [18]. Consistent with previous evidence obtained with xanthine oxidase- or lipoxygenase-induced LPO [21,22], donor NO was found to inhibit photodynamically-initiated LPO in membranes by scavenging free radical intermediates such as LOO^●^ or LO^●^/OLOO^●^ [23].

Another possible fate of photogenerated LOOHs is enzyme-catalyzed 2-electron reduction to redox-inactive lipid alcohols (LOHs). This is a cytoprotective LOOH detoxification process that acts in opposition to damage-enhancing 1-electron LOOH reduction [17]. Glutathione peroxidase-type-4 (GPx4) is now known to detoxify phospholipid hydroperoxides (PLOOHs) as well as cholesterol hydroperoxides (ChOOHs) such as ^1^O_2_-generated 5α-OOH and oxyradical-generated 7α/β-OOH. Initially called phospholipid hydroperoxide glutathione peroxidase (PHGPx) [24], GPx4 is a selenoenzyme (M_r_ ~20 kDa) that uses reduced glutathione (GSH) as a co-substrate. GPx4 appears to be the only enzyme capable of detoxifying PLOOHs and ChOOHs in membrane bilayers or lipoproteins [25]. Glutathione peroxidase-type1 (GPx1), which is much more plentiful in most mammalian cells, can only inactivate PLOOHs after their peroxidized fatty acyl groups are hydrolytically released, e.g., by phospholipases [24,25].

A third possible fate of photogenerated membrane PLOOHs and ChOOHs is translocation to other membranes within a given cell. Lipid trafficking proteins such as SCP-2 and those of the StAR family have been shown to accelerate intermembrane ChOOH translocation [26]. This can result in dissemination of peroxidative damage if hydroperoxide 1-electron turnover occurs more readily in acceptor membrane(s) than in donor(s). On the other hand, if LOOH-detoxifying GPx4 and GSH is more readily available near acceptor compartments, translocation could serve as mode of protection against LOOH damage/cytotoxicity. A similar argument could apply to the relative availability of antioxidants such as low-level NO.

## 4. Role of Endogenous NO in Tumor Resistance to PDT

Two comprehensive studies on how endogenous NO might affect PDT efficacy in vivo were carried out ca. 25 years ago, using various mouse-borne syngeneic tumors sensitized with Photofrin^®^ [27,28]. One of these studies [28] showed that the PDT cure rates for RIF and SCCVII tumors could be significantly improved when an inhibitor of nitric oxide synthase (NOS) activity was administered immediately after irradiation. Tumors with the highest basal rates of NO production were found to respond best, suggesting that NO was playing a protective role [28]. A more recent study involving mouse tumors sensitized with ALA-induced PpIX confirmed these findings [29]. The causal deduction from each of these studies [27,28,29] was basically the same, *viz.* that vasodilation due to endothelium-derived NO was counteracting the known vasoconstrictive effects of PDT [27,30]. Although the earliest phases of this work [27,28]) were ground-breaking in terms of NO’s in vivo ability to antagonize PDT, important questions such as the following were left unsettled: (a) whether the NO derives from tumor cells alone or whether nearby endothelial cells, fibroblasts, macrophages, or neutrophils might contribute; (b) which NOS isoform is most active in any given tumor, i.e., nNOS, eNOS, or iNOS; (c) whether the relevant NOS functions at a pre-existing/constitutive level or is upregulated by PDT stress; and (d) the underlying mechanism(s) by which the NOS-derived NO opposes anti-tumor PDT.

**Figure 2:**
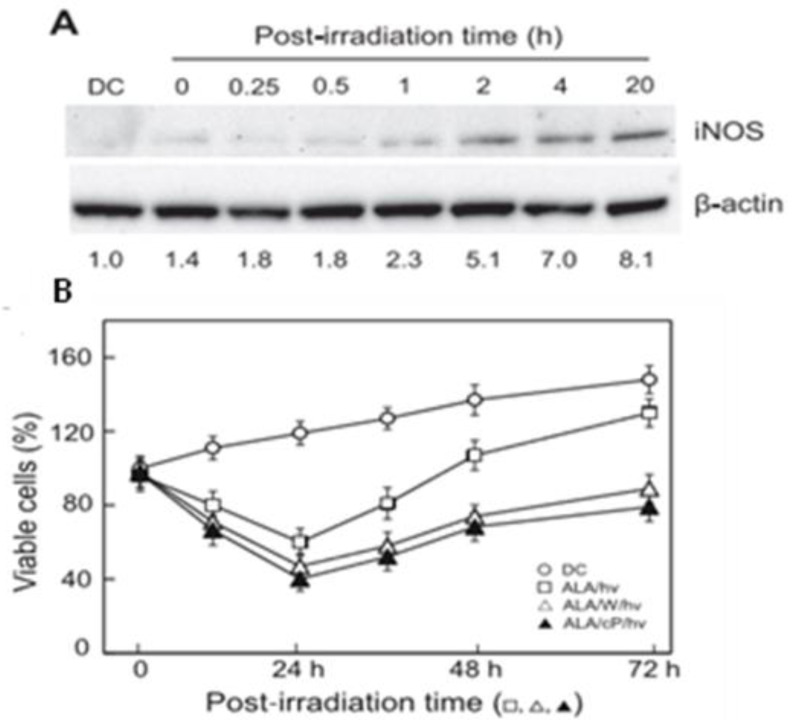
Effects of an ALA/light challenge on post-irradiation iNOS expression, viability, and proliferation of prostate PC3 cells. Cells at ~40% confluence in a serum-free medium were dark incubated with 1 mM ALA for 30 min, then switched to a fresh medium and irradiated with cool white-fluorescent light (fluence ~1 J/cm’). After switching to a 1% serum-containing medium, cells were returned to the incubator. (**A**) iNOS immunoblots on total protein extracts at the indicated post-irradiation times. Numbers below lanes show iNOS band intensity relative to B-actin and normalized to the dark control (DC) up to 20 h. (**B**) Post-irradiation cell viability for 24 h followed by proliferation of surviving (still attached) cells from 24 to 72 h. Cells were ALA/light-treated in the absence vs presence of 25 pM 1400 W or 25 M CPTIO; DC: dark controls (ALA alone). Means + SEM (n = 3). Adapted from Ref. [35].

Early in vitro studies dealing with these questions used human cancer cells (prostate PC3 and breast COH-BR1) photosensitized with ALA-induced PpIX [31]. In addition to confirming that endogenous NO increases cell resistance to photokilling, these studies revealed that iNOS was the principal source of this NO, nNOS and eNOS being relatively unimportant [31]. Of added significance was the finding that the observed hyper-resistance to photokilling was primarily due to photostress-upregulated iNOS rather than pre-existing enzyme [31]. Accordingly, immunoblot analyses showed that the level of iNOS protein increased progressively during post-irradiation incubation of various human cancer lines, including prostate PC3, breast COH-BR1 and MDA-MB-231, and glioblastoma U87 [31]. In PC3 cells, for example, iNOS reached ~8-fold greater than the dark control level after 20 h (Figure 2A) while other NOS isoforms showed no significant changes. Photo-stress upregulation of iNOS was accompanied by a substantial increase in NO steady state level, as detected with a NO fluorophore. At the time, such findings about iNOS/NO were unprecedented for any type of cancer therapy, including chemo- and radiotherapy. Confirmation of iNOS/NO involvement in photokilling resistance was based on evidence such as the following: (a) strong mitigation by a NO scavenger (cPTIO) or by specific inhibitors of iNOS activity (1400 W, GW274150); (b) prevention by siRNA-based iNOS knockdown; and (c) substantial “rescue” of iNOS knockdown cells by a chemical donor (SPNO) that releases NO at low concentrations [31].

As pointed out previously [31,32,33,34], PDT-induced cell killing might actually be enhanced, rather than restricted, by NO if its starting concentration is high enough. The enhancement could be accomplished by using chemical NO donors as PDT adjuvants. Thus, NO can exhibit anti-tumor vs. pro-tumor (“yin” vs. “yang” [5]) properties, depending on its pre-existing local levels. The pro-tumor effects have been the special focus of this review.

The above findings with cultured cancer cells [31] were subsequently corroborated at the in vivo level. Immunodeficient (SCID) female mice carrying MDA-MB-231 breast tumor xenografts were sensitized with ALA-induced PpIX and irradiated with 630 nm LED light [32]. This caused a significant slowdown in tumor growth over a 12-day post-irradiation period. Administration of 1400 W or GW274150 during this period slowed growth even further, consistent with iNOS/NO-dependent resistance; a light-only control was unresponsive to 1400 W. Immunoblot and NO analyses on post-PDT samples revealed a progressive increase in iNOS expression and NO output, each response reaching 5–6-fold over starting level at 6 h post-PDT; thus, in addition to stimulating resistance, NO from overexpressed iNOS after a PDT challenge acted in tumor-supporting fashion, with pre-existing iNOS/NO having no significant effect. Consistently, pro-apoptotic Bax underwent 1400 W-inhibitable downregulation after ALA/light treatment, whereas anti-apoptotic Bcl-xL and Survivin underwent 1400 W-inhibitable upregulation. Collectively, this was the first in vivo evidence for anti-tumor PDT being opposed by the iNOS/NO it induces [32].

## 5. Accelerated Proliferation of PDT-Surviving Cancer Cells: Role of NO

**Figure 3:**
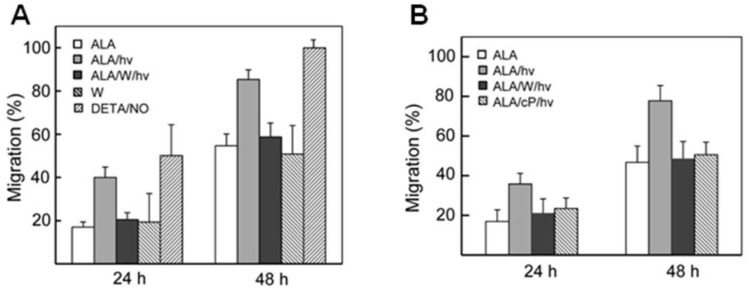
Accelerated migration of photodynamically-stressed prostate cancer PC3 and DU145 cells. Cells of each type at ~90% confluency were pre-incubated with ALA (cf. Figure 1), then either not irradiated (ALA) or irradiated in the absence (ALA/hv) or presence of 1400 W (ALA/Whv). A scratch was made across each cell layer and the extent of gap closure was determined after 24 h and 48 h in the incubator. (**A**) PC3 cells showing results under the above conditions, and also with either W alone or DETA/NO alone (10 µM) present, i.e., without irradiation. (**B**) DU145 cells, showing the effects of including W or PTIO before irradiation. When added before irradiation, agents like 1400 W and PTIO were maintained at the same concentration during subsequent dark incubation. Plotted extents of migration in (**A**,**B**) are means + SD of values obtained from the following relationship: [time-0 gap − time-t gap]/time-0 gap] (n = 6). Adapted from Ref. [35].

This section describes another negative response of cancer cells to photodynamic stress. When surviving (still attached) PC3 cells in vitro were monitored beyond 24 h after ALA/light treatment, a striking observation was made, *viz.* that these cells exhibited a significant growth spurt over at least one additional day. As shown in Figure 3A, cells that had survived photodynamic treatment proliferated faster than dark (ALA-only) controls from 24 h to 48 h, with the calculated rate increase being ~2.7-fold over this period [33]. The large inhibitory effects of 1400 W and cPTIO (Figure 2B) implicated iNOS-derived NO in the rate enhancement. Moreover, the ability of these agents to inhibit cell viability loss over the first 24 h after irradiation confirmed that induced iNOS/NO also had a cytoprotective function in this system [33]. Because most of the ALA-induced PpIX was probably associated with mitochondrial membranes in these cells (see Section 3), PpIX-sensitized chain LPO therein could have played a major cytotoxic role. Overall, results similar to those shown in Figure 1 were obtained with other PDT-stressed cancer lines, e.g., prostate DU145 [33] and glioblastoma U87 [34], suggesting that the observed pro-survival and pro-growth effects of induced iNOS/NO are generally applicable. There needs to be a greater awareness of this possibility among PDT biologists and clinicians.

## 6. Accelerated Migration of PDT-Surviving Cancer Cells: Role of NO

The realization that many cancer cells will proliferate more aggressively if able to withstand a photodynamic challenge stimulated interest in whether this response might be accompanied by more aggressive cell migration. This was first tested using a “wound-healing” or gap-closure assay [36] in which the migration of ALA/light-treated cells into a scratch-voided area on a culture dish was monitored as a function of time after post-irradiation time of incubation at 37 °C. Results obtained with prostate PC3 and DU145 cells are described here. As shown by the bar graph in Figure 3B, surviving PC3 cells that survived ALA/light stress migrated significantly faster than ALA-only controls during post-irradiation dark incubation for a 24 h as well as 48 h period. These findings were confirmed to represent cell migration alone with no significant distortion by cell proliferation occurring simultaneously [35]. The observed ~100% and ~55% faster migration after 24 h and 48 h, respectively, was reduced to nearly the dark control level by 1400 W (Figure 3A). However, 1400 W had no significant effect on the migration rate of cells not exposed to ALA/light, suggesting that basal iNOS/NO was insufficient for stimulation (Figure 2A). Additional support for the stimulatory effects of photostress-upregulated NO was obtained by showing that the migration rate of non-stressed cells could be increased several-fold by an exogenous NO donor, DETA/NO (Figure 3A). These findings with PC3 cells were recapitulated with prostate DU145 cells (Figure 3B), and similar behavior was observed with photostressed glioblastoma U87 cells [34], once again suggesting general applicability to cancer cells, at least at the in vitro level.

## 7. Hyper-Invasiveness of Cancer Cells That Survive a PDT Attack: Involvement of NO

**Figure 4:**
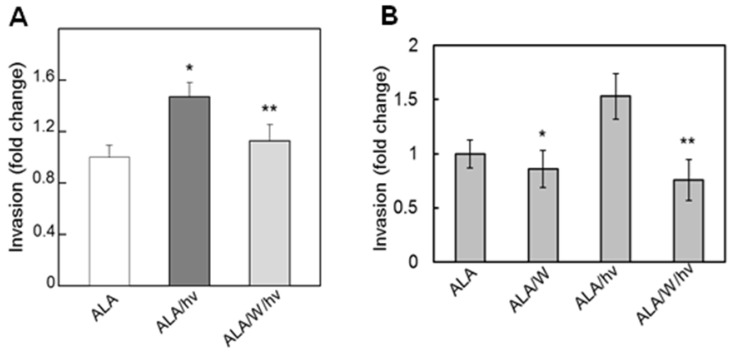
Increased invasiveness of prostate PC3 and glioblastoma U87 cells surviving a photodynamic challenge. Cells were treated with ALA/light in the absence (ALA/hv) vs. presence (ALA/W/hv) of 25 M 1400 W. Immediately after an ALA/light challenge, cells were recovered into a serum-free medium and tested for invasiveness, using CytoSelect^®^ assay modules with basement membrane inserts. The ability of cells to degrade membrane matrix proteins and invade into 10% serum-containing lower chambers was determined. Non-irradiated ALA- and ALA/W-containing controls were run alongside one another. Plotted values are means SD (n = 3) A: PC3 cells, * *p* < 0.05 vs. ALA, ** *p* < 0.05 vs. ALAhv. B: U87 cells: * *p* < 0.05 vs. ALA, ** *p* < 0.01 vs ALA/hv. Adapted from Refs. [34,35].

Observing greater NO-dependent proliferative and migratory aggressiveness of PDT-resistant cells suggested that their ability to initiate metastasis via invasion across an extracellular matrix might respond similarly. For assessing this, a simple and improved version of the Boyden chamber [36] was used, and the ability of cells in a serum-free medium to traverse a Matrigel-infused membrane into a serum-containing medium was assessed. Initial experiments were carried out with ALA/light-treated prostate cancer cells. As shown in Figure 4A, PC3 cells treated with ALA, but not irradiated, exhibited a significant degree of trans-membrane invasiveness over a 48 h period, which represents background activity. Non-ALA-treated cells behaved identically in agreement with the known constitutive invasiveness of PC3 cells [35]. After an ALA/light challenge, recovered surviving cells invaded at least 50% more aggressively than controls over 48 h (Figure 4A). This response was significantly suppressed by 1400 W, indicating that upregulated iNOS/NO had played a key driving role [35]. Similar results were obtained with at least three other cancer lines, breast MDA-MB-231, glioblastoma U251, and glioblastoma U87 [34], the latter being shown in Figure 4B. This raises a concern that any increase in NO-dependent invasiveness occurring during in vivo PDT could promote metastatic tumor progression. This concern remains to be addressed at the clinical PDT level.

Matrix metalloproteinases (MMPs) catalyze the degradation of collagen and other extracellular matrix (ECM) components, thereby playing a key role in cancer cell invasiveness and metastasis [37,38]. Migration/invasion of many tumor cells is stimulated by MMP-9, which becomes activated by proteolytic cleavage of its externalized precursor, pro-MMP-9 [37,38]. In two key studies [34,35], it was found that MMP-9 activity for ALA/light-treated PC3 and U87 cells was consistently much higher than that of dark controls, and that 1400 W or cPTIO blocked this increase. Moreover, a tissue inhibitor of metalloproteinases (TIMP-1) was progressively downregulated in photo-stressed cells, and in 1400 W-inhibitable fashion [35]. This revealed an intricate iNOS/NO-controlled interaction between MMP-9 and TIMP-1 which favored greater invasiveness in stressed cells. Of added importance was the observation that several other proteins that play important roles in tumor progression were upregulated in iNOS/NO-dependent fashion in photo-stressed PC3 and U87 cells, *viz.* integrins α6 and β1, Survivin, and S100A4 [34,35]. Collectively, these findings indicate that the hyper-invasiveness of cells that survive a photodynamic challenge is due to the cooperative action of several different effector proteins. It is important to point out that the studies described [34,35] focused entirely on NO from endogenous or natural iNOS in tumor cells. Thus, they are distinct from many other published studies based on NO from a chemical donor or from transfected iNOS.

## 8. Role of NO in Hyper-Aggressiveness of Bystander Cells after a Targeted Cell PDT Challenge

When malignant tumors are treated with ionizing radiation (e.g., X-rays or γ-rays), heavily targeted cells may send signals to non- or minimally-exposed neighbors, i.e., “bystander cells”. These signals can be transmitted by at least two mechanisms: (a) via gap junctions between cells or (b) via the medium without actual cell contact [39,40]. A variety of bystander effects may be elicited, ranging from DNA damage, impaired damage repair, and apoptotic cell death to enhanced proliferation and motility. Regarding mechanism (b) above, a variety of signaling molecules capable of traversing aqueous media have been identified, e.g., cytokines such as TNF-α and TGF-β, ROS such as O_2_^−●^ and H_2_O_2_, and NO or NO-derived species [41,42,43]. Like O_2_, NO diffuses rapidly and can enter cellular membranes [44]. Unlike H_2_O_2_, NO is not susceptible to scavenging by any known enzymes. Nevertheless, NO has a short aqueous half-life (~1 s), requiring continuous generation by targeted cells for eliciting bystander effects.

**Figure 5:**
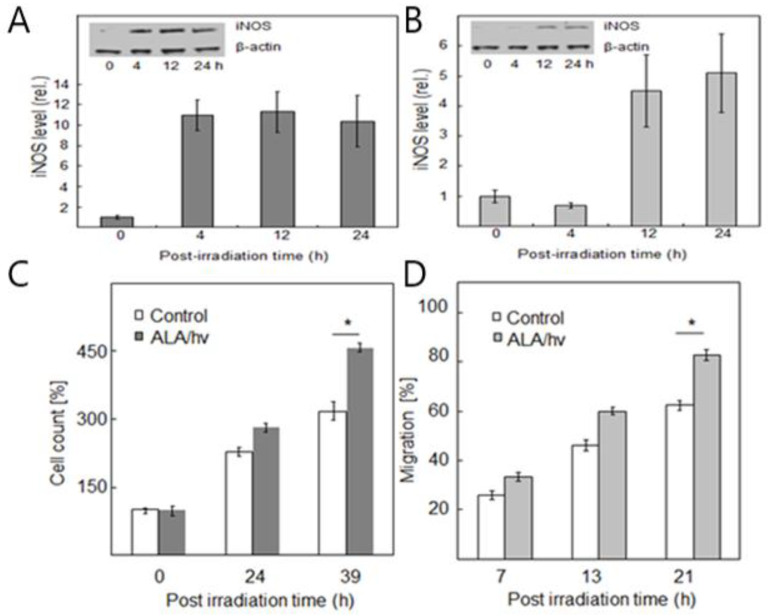
Effects of ALA/light-targeted PC3 cells on non-targeted bystander counterparts. Cells were grown in two separate populations on a large culture dish: one (the smaller) within two silicone-rimmed rings and the other outside the rings. The latter cells, designated as targets, were pre-incubated with ALA, then exposed to LED light (~1 J/cm’). After 2 h in the dark, separating rings were removed and various post-irradiation effects in both populations were evaluated. (**A**) Immunoblot-assessed iNOS level in targeted cells over post-hv time (**B**) Corresponding iNOS level in bystander cells. (**C**) Bystander proliferation during post-hv incubation compared with control lacking ALA. (**D**) Bystander migration during post-h incubation compared with -ALA control. Values in (**A**–**D**) are means + SEM (n = 3–6), * *p* < 0.05 vs. control. Adapted from Refs. [48,49].

That non-ionizing PDT could also induce bystander effects was first described about 25 years ago, but no significant mechanistic information was provided [45]. In subsequent studies by another group [46,47], several mobile signaling species other than NO were proposed, e.g., H_2_O_2_ and LOOHs. Unlike NO, however, these could be susceptible to enzymatic disposal enroute. The first PDT-related study to consider NO as signaling mediator was carried out relatively recently, using a novel silicone-rimmed ring approach for initially separating ALA/light-targeted cells from non-targeted bystanders [48]. Prostate cancer PC3 cells growing on a large culture dish (10-cm diam.) were used exclusively in this initial work. The entire dish was exposed to one dose (~1 J/cm^2^) of broad-band visible light. After 2–3 h of dark incubation, rings were removed, allowing any stress-induced signaling molecules to diffuse from targeted cells to bystanders [48]. There continued to be no physical contact between the two cell populations, so diffusion through the medium was the only means of intercellular communication. As anticipated from previous findings [35], targeted PC3 cells that survived the challenge overexpressed iNOS/NO (Figure 5A) and exhibited a 1400W- and cPTIO-inhibitable growth and migration spurt [48]. Of added great importance, non-stressed bystander PC3 cells also upregulated iNOS/NO (Figure 5B) and began to proliferate (Figure 5C) and migrate (Figure 5D) more rapidly than controls [48]. Upon discovery, these effects were recognized as striking new modes by which NO can antagonize PDT and possibly even promote tumor expansion. Conditioned media from targeted cells failed to elicit these bystander responses, thus ruling out disseminated signaling by relatively long-lived species like cytokines [48]. Therefore, continuously generated NO from overexpressed target cell iNOS appeared to be solely responsible. Several other pro-tumor proteins besides iNOS were upregulated in PC3 bystanders, e.g., cyclooxygenase-2 (COX-2) and protein kinases Akt and ERK1/2, the responsible inducer being NO emanating from targeted cells [48].

Recent studies have compared this bystander behavior of PC3 cells to that of several other human cancer lines, including melanoma BLM, breast MDA-MB-231, and brain U87 [49]. Pre-sensitization with ALA-induced PpIX was the same for all cells, but light fluences were varied so that a uniform, yet modest, target cell kill was attained, e.g., ~25% at 24 h post-irradiation for each cell line. At various post-irradiation times up to 24 h, iNOS levels in targeted cells were assessed by Western blotting, and enhanced bystander proliferation/migration was tracked as a function of maximal iNOS upregulation [49]. For the four lines studies, elevated bystander proliferation or migration rate increased exponentially with maximal targeted iNOS induction. Thus, BLM cells with the lowest induction produced the smallest bystander effects while PC3 cells with the greatest induction produced the largest [49]. Accordingly, the extent of iNOS/NO upregulation in PDT-targeted cells determined the extent of elevated bystander growth/migration aggressiveness, the following order being observed: PC3 > MDA-MB-231 > U87 > BLM [49]. Thus, PC3 cells, which exhibited the greatest iNOS induction, produced the strongest bystander effects, whereas BLM cells with the smallest induction produced the weakest effects. Clearly, therefore, bystander aggressiveness increased proportionately to the extent of iNOS/NO upregulation in targeted cells, the diffusing NO recapitulating its stimulatory effects on neighboring bystanders. This suggests the existence of a NO “feed-forward” process that can propagate through the bystander population, thus promoting overall expansion if occurring in an actual tumor. Thus far, no similar bystander effects based on target cell iNOS/NO induction have been described for any chemo- or radiotherapeutic modality.

## 9. Signaling Events Underlying Increased Cell Aggressiveness after a Photodynamic Challenge

The signaling mechanisms behind many of the described post-PDT phenotypic changes are not completely understood, but studies beginning *ca.* 10 years ago provided some key insights. Experiments with prostate carcinoma PC-3 cells [35] and with glioblastoma U87 cells [50] revealed that transcription factor NF-κB plays a major role in ALA/light-induced iNOS upregulation. In agreement with this, there was a rapid transfer of an NF-κB subunit, p65/Rel A, from the cytosol of photo-stressed cells to the nucleus in preparation for iNOS transcription [35,50]. From non-PDT-based studies by others [51], it was postulated that acetylation of specific lysine residue(s) on p65 is required for stimulating it as a transcriptional co-activator. As supporting evidence, it was found that lysine-310 on p65 of U87 cells became increasingly acetylated (p65-acK310) after the cells were exposed to an ALA/light challenge [50]. Examination of possible upstream signaling events revealed that p65-acK310 formation was dependent on activation of pro-tumor kinases PI3K and Akt, followed by phosphorylation-activation of acetyltransferase p300, in keeping with p65-acK310 elevation. The latter effect was strongly suppressed by C646, an inhibitor of activated p300, thus confirming this enzyme’s role in p65-K310 acetylation. In support of the latter effect, expression of deacetylase sirtuin-1 was downregulated by photodynamic stress while Brd4, an epigenetic reader and iNOS transcriptional co-activator was upregulated [50]. Moreover, PTEN, a known tumor-suppressor and PI3K antagonist, was inactivated via intramolecular disulfide bond formation, which would have supported the sequential activations described above. Expression changes in downstream proteins that regulate invasiveness, e.g., MMP-9, Survivin, and S100A4, were described in Section 7. When considered collectively, these findings reveal a remarkably well-coordinated and cooperative signaling network, beginning with PDT-induced iNOS/NO upregulation, resulting not only in hyper-resistance, but hyper-aggressiveness of stress-withstanding tumor cells.

## 10. Pharmacological Suppression of NO’s Anti-PDT/Pro-Tumor Effects

Pharmacologic inhibition of iNOS activity has been advocated for tumors that significantly rely on iNOS/NO to promote growth and metastatic dissemination. Activity inhibitors such as L-NAME and L-NNA have provided some measure of success with in vitro and in vivo model systems under normal (non-stressed) incubation conditions [52,53,54]. However, these inhibitors are not specific for iNOS and might also affect nNOS or eNOS. Consequently, the possibility of compromising normal physiologic functions such as neuronal activity or blood pressure regulation would be a concern if such agents were considered for clinical use. At least two inhibitors, L-NIL and GW274150, which are highly specific for iNOS, have been safely tested in clinical trials, but these had no relationship to cancer or cancer therapy [55,56].

As pointed out in preceding sections, the ability of iNOS/NO to provide tumor cells with a survival, growth, and migratory advantage is often based on suppression by iNOS-specific activity inhibitors (e.g., 1400 W, GW274150) or by a NO scavenger (e.g., cPTIO). Although iNOS/NO at basal levels may support more aggressive behavior in some cell types, upregulated levels in response to stress-inducing treatments typically produce more dramatic effects. In the case of photodynamic treatment, this was found to be the case at the in vitro (cultured cell) level as well as in vivo (animal model) level [32,34,35]. However, a crucial question is whether the above inhibitors, typically used for diagnostic purposes, would be safe and effective as adjuvants at the clinical PDT level. Thus far, no clinical trials addressing this question have been carried out.

Another possibility for mitigating the negative effects of overexpressed iNOS on PDT would be to suppress its expression at the transcriptional level. Recent studies have shown that accelerated proliferation and migration of surviving U87 glioblastoma cells after an ALA/light challenge could be prevented by JQ1, a bromodomain/extra-terminal domain (BET) inhibitor [57]. JQ1 restricted iNOS generation by binding to the BET domain of Brd4, an epigenetic “reader” protein [58]. Brd4, along with the acetylated p65 subunit of NF-κB, was found to be essential for iNOS transcription in U87 cells [50,58]. When used at 100-fold lower concentration than 1400 W or GW274150, JQ1 was found to be much more effective in suppressing live cell hyper-aggressiveness after an ALA/light challenge [58]. BET inhibitors like JQ1 have been shown to be highly effective in against several different cancers at the in vitro, animal model, and clinical trial levels. Therefore, using BET inhibitors as adjuvants for anti-tumor PDT holds great promise, and we look forward to the first clinical trials for this purpose.

## 11. Antagonistic Effects of iNOS/NO in Other Anti-Tumor Therapies

Although we have emphasized how treatment-imposed resistance reduces PDT effectiveness while stimulating tumor progression, such effects have also been observed in chemotherapy and radiotherapy model systems. For example, during exposure to cisplatin (CP), head-and-neck carcinoma cells upregulated iNOS/NO, followed by Survivin [59]. iNOS inhibition or Survivin knockdown exacerbated CP-induced apoptosis, thereby implicating iNOS-generated NO and Survivin in cell resistance [59]. Other studies with cells from two different cancer types, lung [60] and melanoma [61], showed that endogenous NO could enhance resistance to CP by inhibiting effector proteins via S-nitrosation (SNO) of select residues. More recently, it was reported that iNOS/NO in glioma tumor-associated macrophages (M2-TAMs) induced resistance to CP by inhibiting acid sphingomyelinase (A-SMase), which otherwise stimulates apoptosis [62]. Interestingly, most of the anti-CP NO in this case did not derive from tumor cells themselves, but rather nearby TAMS, which contrasts with the NO described in preceding sections. Regarding radiotherapy, an early study showed that low dose X-radiation (~0.02 Gy) “primed” lung cancer cells to resist killing by subsequent high dose radiation (6 Gy); this resistance was preceded by downregulation of pro-apoptotic p53 and upregulation of iNOS [63]. A recent study on patient-derived pancreatic ductal carcinoma (PDAC) tissue revealed that iNOS/NO was strongly upregulated after radiotherapy—not in PDAC cells per se, but in cancer-associated fibroblasts (CAFs)—and this played a major role PDAC resistance and progression [64]. These findings were subsequently confirmed using mouse-borne orthotopic PDAC tumors [64]. Similar iNOS/NO-mediated resistance to radiation has been observed in various other human cancers in vitro and in vivo [65,66]. In some cases, surviving cells exhibited a significant growth spurt that was similar to that seen in PDT models. Thus, like PDT, other anti-tumor therapies can have negative NO-mediated side effects which require significant mitigation to improve treatment efficacy.

## 12. Summary and Outlook

**Figure 6:**
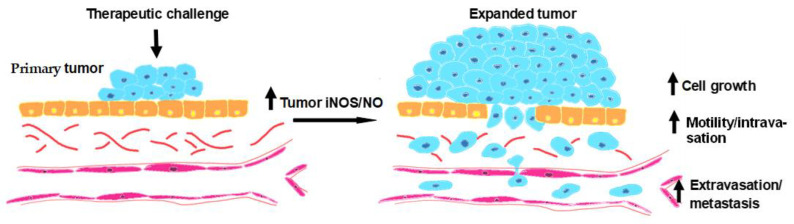
Summary scheme showing how NO from upregulated iNOS in PDT-challenged primary tumors can signal for accelerated proliferation, migration/intravasation, and metastatic invasion/extravasation of surviving tumor cells.

We have discussed how low levels of iNOS-derived NO can antagonize anti-tumor PDT in various ways, e.g., imposing greater tumor cell resistance to photokilling, and stimulating proliferation, migration, and invasiveness of cells that can withstand the photodynamic challenge. [31,32,33,34,35]. The scheme shown in Figure 6 illustrates these anti-PDT effects of upregulated iNOS/NO. Such effects can also occur in non- or minimally-stressed bystander cells due to uptake of NO diffusing form heavily PDT-targeted cells in any given tumor [49]. Although we have focused here on various manifestations of iNOS/NO antagonism to PDT, similar negative effects can also occur in other anti-tumor modalities such as chemo- and radiotherapy. As indicated in this review, the low-level NO involved in the PDT-induced effects typically derives from stress-upregulated iNOS rather than enzyme at preexisting levels. Similar responses have been observed for other anti-tumor modalities, e.g., cisplatin-based chemotherapy, but in the case of PDT, at least, dependency on NO from overexpressed rather than preexisting iNOS is not well-recognized. The steady state levels of this NO after a PDT challenge are much lower than those from macrophage iNOS during an immune response [5], but how the former are regulated is not yet clear. Concerns about greater migratory and invasive aggressiveness of PDT-surviving or bystander cells can be alleviated by pharmacologic intervention with inhibitors of iNOS activity or transcription. In the latter category, recent findings with BET inhibitor JQ1 [58] suggest that at the clinical level, BET inhibitors will be more effective than iNOS activity inhibitors, and at much lower doses. We look forward to the first clinical trials involving use of BET inhibitors as PDT adjuvants.
